# Correlation of Neuroanatomical Structures Related to Speech in Cerebral Palsy Patients Aged 0–17: A Retrospective MRI Study

**DOI:** 10.3390/children12020249

**Published:** 2025-02-19

**Authors:** Erhan Berk, Rümeysa Üzümcüoğlu, Feyza İnceoğlu, Merve Aydın, Muhammed Furkan Arpacı, Ahmet Sığırcı, Hıdır Pekmez

**Affiliations:** 1 Department of Pediatrics, Faculty of Medicine, Malatya Turgut Özal University, 44210 Malatya, Türkiye; erhan.berk@ozal.edu.tr; 2 Department of Anatomy, Institude of Graduate Science, Malatya Turgut Özal University, 44210 Malatya, Türkiye; 36223634001@ozal.edu.tr; 3 Department of Biostatistics, Faculty of Medicine, Malatya Turgut Özal University, 44210 Malatya, Türkiye; feyza.inceoglu@ozal.edu.tr; 4 Department of Anatomy, Faculty of Medicine, Malatya Turgut Özal University, 44210 Malatya, Türkiye; merve.aydin@ozal.edu.tr (M.A.); furkan.arpaci@ozal.edu.tr (M.F.A.); 5 Department of Radiology, Turgut Özal Medical Center, İnönü University, 44000 Malatya, Türkiye; ahmet.sigirci@inonu.edu.tr

**Keywords:** cerebral palsy, speech related brain regions, volume, MRI

## Abstract

**Background/Objectives**: Cerebral Palsy (CP) is a non-progressive clinical condition characterized by secondary issues, including speech impairments. Our study aims to evaluate the volumes of brain areas related to speech in patients diagnosed with CP between the ages of 0–17. **Methods**: this study includes the images of 84 children: 42 in the control group who applied to the hospital between the specified dates and were reported as healthy by MRI from the patient records, and 42 patients with CP. **Results**: in the CP group, white and gray matter, cerebrum, cerebellum, thalamus, lobus frontalis, lobus temporalis, lobus parietalis, lobus insularis, gyrus cinguli, and nuclei basales volumes were observed to decrease statistically significantly compared to the control group (*p* ˂ 0.001). **Conclusions**: we found a significant decrease in the volumes of speech-related brain areas in CP patients, indicating that CP can significantly impact the brain’s speech-related regions.

## 1. Introduction

Cerebral Palsy (CP) is a non-progressive clinical condition resulting from damage to the developing immature brain. It is characterized by secondary issues including movement and balance disorders, speech impairments, cognitive deficits, as well as hearing and vision loss [1,2,3]. During pregnancy or in the first years of life, conditions such as hypoxia, infection, stroke, hypotension, premature white matter disease (periventricular leukomalacia), or trauma can lead to CP [4]. It is estimated that around 80% of cases of CP occur during pregnancy. CP often leads to comorbidities such as prenatal brain developmental disorders, epilepsy, communication disorders, dysphagia, and cognitive impairment [5].

Approximately 20% of children with CP have motor speech disorders [6]. Speech and language difficulties can arise from issues related to speech-motor control, cognitive function, language processing, sensation/perception, or a combination of these factors [7]. It has been reported that even children with CP who do not have dysarthria may experience delayed speech development compared to their peers [6].

The brain’s speech-related areas are linked to various brain lobes. The motor speech center is situated in the frontal lobe. Additionally, there are regions responsible for sensory-motor mapping during speech, the utilization of inner speech mechanisms during language encoding, word meaning differentiation, and speech rhythm [8,9]. There is a secondary sensory area in the parietal lobe responsible for speech perception, comprehension, word meaning matching, and reading comprehension [10]. In the temporal lobe, word production and comprehension occur on the left side, while important areas for awareness of the melody and rhythm of speech are located on the right side. There are also areas responsible for the formation of gestures in speech and the auditory perception of both speech and non-speech sounds [11,12,13]. The cerebellum is involved in processing emotional speech rhythms during verbal communication, as well as in tasks related to verbal fluency, word retrieval, and reading and writing [14,15]. In addition, the basal nuclei are responsible for encoding rhythmically designed sound-related structures and using them with motor programs in speech-motor learning [16,17]. Studies have indicated a significant difference in brain region volumes for individuals with speech impairments [1,2,3,4,5].

Although speech problems in children with CP have been recognized for years, no study has been published on the potential effects of this condition on the brain. Our study aims to evaluate the volumes of areas related to speech in patients diagnosed with CP between the ages of 0 and 17.

## 2. Materials and Methods

This research was conducted in the Radiology Department of Turgut Özal Medical Center. From 2019 to 2022, we examined the image records of patients diagnosed with Cerebral Palsy. Ethical approval was obtained with the decision of the Malatya Turgut Özal University Non-Invasive Clinical Research Ethics Committee, dated 16 May 2023, and numbered B.169.

This study includes the images of 84 children, 42 in the control group who applied to the hospital between the specified dates and were reported as healthy by MRI from the patient records, and 42 patients with CP. Children who had head trauma and cranium operations were not included in the study. There was no statistical difference between groups according to gender (×2: 0.001, *p*: 1.000).

The MRIs were performed by taking axial T1-weighted images on SIEMENS Amira (Enlargen, Germany) 1.5 Tesla device. MRI protocol: 3D T1-MPRAGE Repetition time (TR): 2200 ms, Echo time (TE): 2.79 ms; Flip angle (declination): 8°; Field of View (FOV): 250 mm; number of sections: 192; section thickness: 1 mm; matrix: 205 × 320. The acquired images were analyzed using the volBrain program.

VolBrain 1.0 is a free, automated online system (http://volbrain.upv.es/ (accessed on 6 June 2023)) that allows for the measurement of brain volumes without requiring human intervention. It conducts a volumetric analysis of T1-weighted images autonomously. The volBrain pipeline consists of a series of image processing tasks designed to enhance the input image quality and align them with a particular geometric and intensity standard, specifically the one used for manually labeled training templates, before segmenting the various structures and tissues of interest [18].

In our study, the volumes of the lobus frontalis, parietalis, temporalis, insularis, nuclei basales, thalamus, gyrus cingulate, cerebellum, total cerebrum, white matter, and gray matter were analyzed using volBrain.

### Statistical Analysis

The study’s sample size was determined through a power analysis utilizing G*power 3.1 software, resulting in a minimum requirement of 80 participants (40 per group) [19]. The research data were analyzed using Statistical Program in Social Sciences (SPSS 25), including the calculation of descriptive statistics such as number, percentage, mean, standard deviation, median, and min–max. The independent *t*-test was employed to compare independent groups.

An ANCOVA analysis was applied to purify the effect of the covariate variable, and the results were given.

## 3. Results

There was no statistical difference between the groups according to gender (*p* > 0.05) (Table 1). However, there was a statistical difference between the groups according to age (*p* < 0.05). The data showed a homogeneous distribution according to gender. However, since a homogeneous distribution was not provided according to age, age was included in the model as a covariate variable with ANCOVA analysis, and the changes in the variables in the groups were controlled.

In the CP group, the volumes of white matter, gray matter, cerebrum, cerebellum, and thalamus were found to have decreased significantly when compared to the control group (*p* ˂ 0.001) (Table 2).

As a result of including age as a covariate variable in the model, it was found that age had an effect on the cerebellum (*p* < 0.05). It was observed that the differences between the groups in white matter, gray matter, cerebrum, and thalamus measurements were not affected by age (*p* > 0.05).

When the volumes of the speech-related areas in the frontal lobe, temporal lobe, parietal lobe, insular lobe, and cingulate gyrus of the patient and control groups were compared, it was observed that the volumes statistically decreased in the CP group (*p* ˂ 0.001) (Table 3).

Including age as a covariate variable in the model revealed that the differences between the groups in the measurements of volumes of the speech-related areas in the frontal lobe, temporal lobe, parietal lobe, insular lobe, and cingulate gyrus were not affected by age (*p* > 0.05).

In the CP group, the volumes of the basal nuclei (basal forebrain, caudate, pallidum, and putamen) were observed to decrease statistically significantly compared to the control group (*p* ˂ 0.001) (Table 4).

As a result of including age as a covariate variable in the model, it was found that age had an effect on the basal forebrain (*p* < 0.05). It was observed that the differences between the groups in the nuclei basales (caudate, pallidum, and putamen) volume measurements were not affected by age (*p* > 0.05).

## 4. Discussion

One key neurological finding in CP is brain atrophy [20]. Given the relationship between brain volumes and neurological functions, it can be inferred that speech disorders and brain volumes may be correlated in cases of CP.

Speech difficulties are common in individuals with CP, occurring in approximately 21% of patients. CP can impact various aspects of speech production, such as laryngeal, velopharyngeal, and articulatory movements, as well as breathing [3]. Previous studies have measured brain volume in various speech disorders and Cerebral Palsy (CP). However, the specific examination of speech areas in individuals with CP has not yet been explored [21,22,23,24].

Lin et al. conducted studies to assess brain volumes in individuals with hearing loss and found a significant decrease in the volume of the superior temporal gyrus, medial temporal gyrus, superior parietal lobe, and cingulate gyrus. Additionally, they reported a substantial reduction in total brain volume [25]. According to Rudner et al.’s research, it was found that individuals with poorer speech reception threshold in noise issues exhibited a reduction in the volumes of the insular cortex and gyrus precentralis [26]. Similarly, significant volume loss was detected in these regions in children with CP.

In the research conducted by Beal et al. on stuttering children, they observed that there was a decrease in volume within the inferior frontal gyrus and the putamen, pars opercularis, pars orbitalis, and pars triangularis. They also observed increased volume in the gyrus postcentralis, superior temporal gyrus, and inferior parietal lobule [27]. Preston et al. conducted a study that found a significant increase in volume in specific areas of the brain, including the superior temporal gyrus, planum temporale, supramarginal gyrus, planum polare, and transverse temporal gyrus, in children who exhibited speech errors when compared to a control group [28]. According to Pigdon et al.’s study, individuals with developmental speech and language disorders exhibited an increase in the volumes of several brain areas linked to speech and language, including the superior temporal gyrus, inferior frontal gyrus, putamen, gyrus supramarginalis, cerebellum, nucleus caudatus, and putamen, when compared to the control group [29]. Beal, Preston, and Pigdon conducted a series of studies on children with speech disorders and stuttering. The participants in these studies were otherwise healthy, with the exception of speech errors. Through fMRI analysis, it was observed that during voluntary word repetition, the superior temporal gyrus and supramarginal gyrus exhibited higher than normal levels of activity [30]. These studies have shown that there is an increase in volume in the STG, planum polare, planum temporale, transverse temporal, and supramarginal gyrus in children who stutter and make speech errors. This increase in volume is due to the repetitive nature of their speech errors. However, in our study, we observed a volume loss in these regions in children with CP. This volume loss may be attributed to their inability to perceive speech sounds accurately, which leads to errors in speech production without their awareness. Unlike children who stutter and repeat words or sounds, children with CP are unaware of their speech errors and do not repeat them [31]. In addition, children with CP may have different problems that may cause speech disorders, such as dysphagia, drooling problems, swallowing and chewing problems, involuntary muscle contractions, and intellectual disability, which may also cause this difference [32]. In our study, we believe that speech problems in patients with CP are not only caused by volumetric changes in the speech regions but also that decreases in volume in other regions may influence this situation.

In a study on brain aging and speech production, the importance of the nucleus caudatus, basal ganglia, supplementary motor cortex, and cerebellum in speech was reported [33]. In our study, a significant decrease was found in the volumes of these regions.

Kulak et al. [22] found that gray matter, brain, and cerebellum volumes decreased in CP patients. In the study of Spencer et al. [21], a significant decrease was found in the volumes of the putamen, pallidum, and thalamus. Other studies in the literature have reported a decrease in cerebellum volume in children with CP [23,24]. In our study, we found that there was a significant decrease in brain volumes in various regions, which is consistent with the findings of previous studies. Apart from those regions, we also observed significant volume loss in the frontal, temporal, and parietal lobes, as well as the gyrus of these regions, the insula, and the gyrus cinguli.

Studies have reported that volume losses in certain areas of the brain are associated with deterioration in the functions of those areas [26,34]. Also it has been reported that volume changes in speech-related areas have an effect on different stages of speech production, such as reaction time and clarity of articulation [33]. For this reason, it is likely that cerebral palsy patients in whom we find volume differences in speech-related regions may be experiencing functional speech problems. In the literature that investigates brain volume in individuals with cerebral palsy, researchers typically measure the volumes of various brain regions, including total gray and white matter, as well as specific structures such as the cerebellum, pallidum, thalamus, nucleus caudatus, and putamen [21,22,23,24]. Our study aimed to evaluate the volumes of speech-related areas in patients with CP, which has not been extensively studied in the literature.

## 5. Conclusions

We observed a significant reduction in the volumes of these specific areas in patients with CP, indicating that CP can have a substantial impact on the brain’s speech-related regions. These findings could provide valuable insights into the neurological underpinnings of speech impairments in CP patients and could aid in the development of targeted interventions to improve speech outcomes.

## 6. Limitations

One of the limitations of our study is that the measurements were compared without distinguishing among the various subtypes of cerebral palsy. Additionally, functional speech assessments were not conducted on patients with cerebral palsy, and brain volumes were not correlated with functional speech impairments. Furthermore, the volumes of the right and left hemispheres were not evaluated in our research. Given that the dominant cerebral hemisphere is particularly impacted in cases of speech disorders, it is crucial to assess the hemispheres separately. To enhance the effectiveness of future studies, it would be advantageous to complement volumetric analyses with functional assessments. Specifically, incorporating functional tests related to speech alongside volumetric examinations in speech-related areas may yield greater support for the findings.

## Figures and Tables

**Table 1:** Comparison of gender by groups.

**Variable**	**Groups**	**n %**	**Groups**		**Total**	**ꭓ^2^**	***p***
			**Control**	**CP**			
Sex	Female	n	42	42	84	0.001	1.000
		%	50.0%	50.0%	50.0%		
	Male	n	42	42	84		
		%	50.0%	50.0%	50.0%		
**Variable**		**Gruplar**	**Mean ± sd**	**(Min–Max)**		**t**	***p* Value**
Age		Control	7.12 ± 1.64	(3–10)		144.557	0.001 *
		CP	3.61 ± 2.12	(2–11)			

n: number; %: percentage; ꭓ^2^: chi-square test value; sd: standard deviation; t: independent *t*-test; * *p* < 0.05; there was a statistical difference between the groups (*p* < 0.05).

**Table 2:** Comparison of white matter, gray matter, cerebrum, cerebellum and thalamus volume measurements by groups.

Variables (cm^3^)	Groups	Mean ± sd	t	*p*	F	*p*
White Matter	Control	433.8 ± 54.15	92.973	0.001 *	54.342	0.001 *
	CP	316.25 ± 98.06				
Gray Matter	Control	769.69 ± 94.09	132.454	0.001 *	83.385	0.001 *
	CP	539.18 ± 158.27				
Cerebrum	Control	1187.47 ± 1089.18	12.53	0.001 *	8.559	0.001 *
	CP	759.69 ± 223.22				
Cerebellum	Control	123.52 ± 13.7	11.959	0.001 *	0.667	0.415
	CP	88.96 ± 22.69				
Thalamus	Control	13.53 ± 2.4	100.823	0.001 *	56.852	0.001 *
	CP	9.13 ± 3.24				

sd: standard deviation; t: independent *t* test; CP: Cerebral Palsy; * *p* < 0.05: there was a statistical difference between the groups (*p* < 0.05); F: ANCOVA test value; covariate variable: age.

**Table 3:** Comparison of volume measurements of speech-related areas in lobus frontalis, lobus temporalis, lobus parietalis, lobus insularis and gyrus cinguli according to groups.

Variables (cm^3^)	Groups	Mean ± SD	t	*p*	F	*p*
Frontal lobe	Control	197.89 ± 27.59	94.076	0.001 *	53.395	0.001 *
	CP	142.79 ± 44.27				
Opercular inferior frontal gyrus	Control	7.11 ± 1.48	62.798	0.001 *	37.082	0.001 *
	CP	5.02 ± 1.92				
Orbital inferior frontal gyrus	Control	3.42 ± 0.9	54.215	0.001 *	31.695	0.001 *
	CP	2.34 ± 0.99				
Triangular inferior frontal gyrus	Control	8.18 ± 1.39	83.107	0.001 *	43.628	0.001 *
	CP	5.67 ± 2.12				
Precentral gyrus	Control	26.53 ± 3.97	58.754	0.001 *	33.969	0.001 *
	CP	20.14 ± 6.53				
Superior frontal gyrus	Control	31.54 ± 5.38	70.945	0.001 *	38.821	0.001 *
	CP	23.39 ± 7.11				
Supplementary motor cortex	Control	11.29 ± 2.89	29.42	0.001 *	14.599	0.001 *
	CP	8.88 ± 2.85				
Temporal lobe	Control	127.83 ± 16.13	140.116	0.001 *	91.441	0.001 *
	CP	86.48 ± 27.76				
Planum polare	Control	4.38 ± 0.86	47.889	0.001 *	27.809	0.001 *
	CP	3.25 ± 1.24				
Planum temporale	Control	4.33 ± 1.08	76.436	0.001 *	49.330	0.001 *
	CP	2.68 ± 1.34				
Inferior temporal gyrus	Control	25.96 ± 3.94	99.671	0.001 *	64.414	0.001 *
	CP	18.34 ± 5.77				
Middle temporal gyrus	Control	34.56 ± 4.78	146.615	0.001 *	95.143	0.001 *
	CP	22.62 ± 7.67				
Superior temporal gyrus	Control	17.52 ± 2.5	95.024	0.001 *	60.463	0.001 *
	CP	12.54 ± 3.96				
Transverse temporal gyrus	Control	2.87 ± 0.66	66.608	0.001 *	35.814	0.001 *
	CP	1.94 ± 0.82				
Parietal lobe	Control	126.45 ± 21.38	88.895	0.001 *	50.545	0.001 *
	CP	89.71 ± 28.61				
Angular gyrus	Control	27.05 ± 5.34	119.49	0.001 *	69.362	0.001 *
	CP	17.31 ± 6.18				
Postcentral gyrus	Control	23.44 ± 4.49	46.456	0.001 *	23.283	0.001 *
	CP	17.64 ± 6.38				
Superior parietal lobule	Control	26.29 ± 5.35	50.649	0.001 *	26.329	0.001 *
	CP	19.82 ± 6.4				
Supramarginal gyrus	Control	20.36 ± 4.06	78.754	0.001 *	46.853	0.001 *
	CP	14.32 ± 4.75				
Anterior insula	Control	8.25 ± 1.35	91.027	0.001 *	53.163	0.001 *
	CP	5.47 ± 2.3				
Posterior insula	Control	4.44 ± 0.83	83.169	0.001 *	34.904	0.001 *
	CP	2.95 ± 1.26				
Anterior cingulate gyrus	Control	12.7 ± 2.49	90.103	0.001 *	44.584	0.001 *
	CP	8.2 ± 3.57				
Middle cingulate gyrus	Control	10.86 ± 1.69	61.611	0.001 *	54.773	0.001 *
	CP	8.01 ± 2.86				
Posterior cingulate gyrus	Control	10.54 ± 1.55	74.684	0.001 *	51.685	0.001 *
	CP	7.66 ± 2.63				

sd: standard deviation; t: independent *t*-test; CP: Cerebral Palsy; * *p* < 0.05: there was a statistical difference between the groups (*p* < 0.05); F: ANCOVA test value; covariate variable: age.

**Table 4:** Comparison of nuclei basales volumes by groups.

Variables (cm^3^)	Groups	Mean ± sd	t	*p*	F	*p*
Basal forebrain	Control	0.46 ± 0.12	13.753	0.001 *	2.928	0.089
	CP	0.38 ± 0.15				
Caudate	Control	5.63 ± 1.23	104.812	0.001 *	65.888	0.001 *
	CP	3.17 ± 1.82				
Pallidum	Control	2.47 ± 0.55	152.225	0.001 *	104.001	0.001 *
	CP	1.3 ± 0.68				
Putamen	Control	7.54 ± 1.51	151.014	0.001 *	100.996	0.001 *
	CP	4.38 ± 1.82				

sd: standard deviation; t: independent *t*-test; CP: Cerebral Palsy; * *p* < 0.05: there was a statistical difference between the groups (*p* < 0.05). F: ANCOVA test value; covariate variable: age.

## Data Availability

All the data sets used for the study are available from the corresponding author upon reasonable request due to privacy concerns regarding personal information and ethical considerations in ensuring participant confidentiality.
